# Clinical Characteristics of Older Adults Living in Foster Families in the French West Indies: Baseline Screening of the KArukera Study of Aging in Foster Families (KASAF) Cohort

**DOI:** 10.1093/geroni/igae063

**Published:** 2024-06-28

**Authors:** Denis Boucaud-Maitre, Roxane Villeneuve, Christine Rambhojan, Nadine Simo-Tabué, Nathalie Thibault, Leila Rinaldo, Jean-François Dartigues, Moustapha Dramé, Hélène Amieva, Maturin Tabué-Teguo

**Affiliations:** DRCI, Centre Hospitalier Le Vinatier, Bron, France; Equipe EPICLIV, Université des Antilles, Fort-de-France, Martinique, France; Inserm U1219 Bordeaux Population Health Center, University of Bordeaux, Bordeaux, France; DRCI, Centre Hospitalo-Universitaire de Guadeloupe, Pointe-à-Pitre, Guadeloupe, France; Service de Gériatrie, Centre Hospitalo-Universitaire de Martinique, Fort-de-France, Martinique, France; DRCI, Centre Hospitalo-Universitaire de Guadeloupe, Pointe-à-Pitre, Guadeloupe, France; DRCI, Centre Hospitalo-Universitaire de Guadeloupe, Pointe-à-Pitre, Guadeloupe, France; Inserm U1219 Bordeaux Population Health Center, University of Bordeaux, Bordeaux, France; Service de Gériatrie, Centre Hospitalo-Universitaire de Martinique, Fort-de-France, Martinique, France; Inserm U1219 Bordeaux Population Health Center, University of Bordeaux, Bordeaux, France; Equipe EPICLIV, Université des Antilles, Fort-de-France, Martinique, France; Service de Gériatrie, Centre Hospitalo-Universitaire de Martinique, Fort-de-France, Martinique, France

**Keywords:** Caribbean, Dementia, Dependency, Foster family

## Abstract

**Background and Objectives:**

Foster families for older adults could represent a transitional or alternative model to nursing homes. The aim of this study was to describe the clinical characteristics of older adults in foster families and to compare them with those of residents in nursing homes in French West Indies.

**Research Design and Methods:**

This study is a cross-sectional analysis of the KArukera Study of Aging in Foster Families (KASAF) cohort. Sociodemographic and clinical characteristics were extracted. Dependency was assessed using the Activities of Daily Living (ADL) scale and cognition using the Mini-Mental State Examination (MMSE) scale. Age, gender, ADL, and MMSE scores were compared with nursing home residents from a twin study of KASAF (*n* = 332).

**Results:**

A total of 107 older adults (mean age 81.8 years; 61.7% women) were recruited in 56 foster families between September 2020 and May 2021. In all, 25.5% had diabetes mellitus and 45.8% suffered from hypertension. The mean MMSE score was 9.3 ± 10.1 and 76.0% had major cognitive impairment (MMSE score <18); 12.5% were diagnosed with Parkinson’s disease, and 42.0% of the residents were confined to bed or in a wheelchair, with a mean ADL score of 1.5 ± 1.8. Almost all the residents (96.3%) benefited from a medical follow-up by a nurse who visited once or twice a day. Compared to older adults living in nursing homes, those in foster families were more frequently women (61.7% vs 49.4%) and had lower ADL score (1.5 vs 2.4) and lower MMSE score (9.3 vs 11.3).

**Discussion and Implications:**

The clinical profile of foster families’ residents was quite similar to that of nursing home residents in terms of demographics, dementia, and dependency. Foster families might represent an interesting strategy to address the unmet clinical and social needs of dependent older adults, especially in countries where nursing homes are not sufficiently developed.

**Clinical Trials Registration Number:**

NCT04545775

**Translational Significance:** In high-income countries, the care of dependent older adults relies primarily on nursing homes. The present study examines a model of a foster family for older adults with paramedical supervision in Guadeloupe, French West Indies. We observed that the clinical profile of older adults in these foster families seems to be similar to that observed in nursing homes. Foster families might have a place in the evolving organization of care in many countries, to address the unmet needs of older adults.

With the aging of the population, managing the dependency of older people is a global public health issue. Every country in the world face many challenges in meeting the long-term care needs of their aging populations are numerous. Most of today’s older adults live in developed countries. However, many low- and middle-income countries will experience a greater increase of dependent people than high-income countries. The total number of dependent older adults will almost double by 2050 (Prince et al., 2013) and projections suggest that nearly 80% of the world’s older population will live in developed countries in 2050 (United Nations, 2017). In high-income countries, policies for the care of dependent people focus mainly on home care, support for caregivers, or institutionalization. In low- and middle-income countries in Africa or South America, nursing homes are limited (Wachholz et al., 2021), due to the lack of existing structures and the cost involved. Although some private long-term facilities exist, they are only accessible to a very small minority of older adults. As a result, families are often the main source of care for older adults who can no longer live independently (Scheil-Adlung, 2015). There is a need to develop alternative models for dealing with older adults, depending on their needs at the individual, community, and societal levels. Indeed, given the diversity of people’s aging trajectories, and the diversity of their social and economic profiles, it is becoming increasingly clear that proposing a single model of accommodation (nursing home) for dependent older adults is inappropriate. These models must be able to meet the individual needs of older people, depending on their physical and cognitive functions, their psychological state, their comorbidities, and the country’s social and economic environment.

Among these models, foster families for older adults may represent an interesting strategy, which has been little studied and assessed in the scientific literature (Boucaud-Maitre, Cesari et al., 2023). Each foster family is responsible for one to three people aged 60 years or over; the family provides a room in the house for each older adult and offers them meals and activities. Although foster families for older adults have long existed in many countries (officially or not), only three American studies conducted more than 30 years ago assessed their effectiveness, without providing any answers in terms of mortality or hospitalizations (Young et al., 2017). This model of care has developed considerably in Guadeloupe (French West Indies) over the last 20 years. Guadeloupe is a French overseas territory in the Caribbean. In this territory, 90% of the population is of African descent. The medical and cultural realities are quite different from those in mainland France. The epidemiology of the older adults in this territory presents a number of particularities that overlap with issues affecting both high- and middle-income countries. The area is experiencing an accelerated aging of the population, with the proportion of people aged over 60 years already reaching 29% of the total population (INSEE, 2023). There are also strong specificities in terms of the prevalence of certain diseases, in particular hypertension, diabetes (Filipovic-Pierucci et al., 2016), or Parkinson’s disease (Lannuzel et al., 2007), specific genetic risk factors, and socioeconomic characteristics or access to healthcare. The social indicators show that the standard of living in the overseas territories is lower than in mainland France. In 2018, the poverty rate was 34% compared to 14% in mainland France (INSEE, 2022) and the rate of high poverty level was 12% compared to 2.1%. Guadeloupe also faces a shortage of doctors and care facilities for dependent older people, with 35.6 places in nursing homes per 1,000 people over 75, compared with 122.6 in mainland France. Moreover, the average cost of nursing homes in the French West Indies is one of the most expensive in France, ranging from 3,000 to 4,500 euros per month, compared with an average price of 1,950 euros in mainland France, for a population that is extremely vulnerable economically. Consequently, a proactive policy has been implemented to promote foster families for older adults, due to the lack of alternatives and probably to anthropological social factors with the central role of the family. The local authorities pay for the accommodation for the older adults and a 54-hr training course is required to become a foster career. Foster families are presented locally as an alternative to nursing homes, that is, as a suitable solution for dependent older people, but there is no epidemiological data to confirm this.

In this context, a prospective study was conducted in foster families (KASAF, KArukera Study of Aging in Foster Families) in Guadeloupe. KASAF study followed the health trajectory of residents in foster family over a 1-year period in terms of hospitalizations, mortality, and geriatric syndromes. This first report describes the study population at inclusion. The aim of this study was to identify the clinical specificities of residents in foster families. The secondary objective was to compare the demographic characteristics, level of autonomy, and cognition of residents in foster families with residents in French West Indies nursing homes.

## Method

### KASAF Study

This study is a cross-sectional analysis of the KASAF study, examining the baseline characteristics of older adults in foster families and the sociodemographic characteristics of their family caregivers. The protocol has been published (Boucaud-Maitre, Villeneuve et al., 2023) and is registered on ClinicalTrials.gov (NCT04545775). The KASAF study was approved by the French Sud Méditerranée III Ethics Committee Ethics on July 1, 2020. Briefly, KASAF is a multicenter, prospective, observational 1-year follow-up study designed to investigate the care pathways of older adults with dependency in foster families (KASAF). We have collected the socio-medico-economic data of older residents in foster families over a year. Data were collected through face-to-face interviews with the participants and their caregivers at baseline and after 6 and 12 months and though phone interviews after 3 and 9 months. To be eligible, residents had to be over 60 years old, living in a foster family in Guadeloupe, and benefit from the French social security.

Recruitment opened in September 2020 and ended in May 2021, with 107 older people recruited and 56 respective foster caregivers. The 1-year follow-up ended in June 2022.

For this study, we extracted the sociodemographic characteristics of the family caregivers and the clinical baseline characteristics of residents from KASAF: age, gender, marital status, duration of stay in foster families, number of paramedical visit, comorbidities, alcohol and tobacco use, dependency, cognition, depression, physical function, status of nutrition, and quality of life.

Sociodemographic and comorbidity data were collected by nurses and geriatricians during on-site visits with residents and their foster caregivers. The patient form listed the major medical comorbidities (e.g., diabetes, hypertension, stroke, cardiovascular diseases, chronic obstructive pulmonary disease, asthma, arthritis, osteoporosis, cancer, Parkinson’s disease, Alzheimer’s disease and other dementias, depression, vision, and hearing difficulties).

The level of dependency in activities of daily living (ADL) was assessed using the Katz scale (Katz et al., 1963). This six-item scale assesses six basic ADLs: bathing, toileting, transferring, eating, dressing, and incontinence. For each activity, a score of 1 indicates complete autonomy, a score of 0.5 indicates partial autonomy, and a score of 0 indicates complete dependency. The level of independence in instrumental ADL (IADL) was assessed using the Lawton IADL scale (Lawton et al., 1969). The Lawton IADL scale measures eight items: using the telephone, shopping, preparing food, housekeeping, doing laundry, using transportation, handling medications, and handling finances. The total score ranges from 0 (total dependency) to 8 (total autonomy). Finally, the level of dependency at entry and inclusion was assessed with the Autonomy, Gerontology Iso-Resources (AGGIR) questionnaire. In France, the AGGIR questionnaire has been developed by the public authorities to quickly assess the level of dependency of older adults and to assign a dependency group, ranging from GIR = 0 (bedridden, totally dependent) to GIR = 6 (no help required, the person is autonomous in daily activities). The GIR scores have been grouped into three classes: high dependency (GIR 1 or 2), moderate dependency (GIR 3 and 4), and low dependency (GIR 5 or 6).

The level of cognitive decline was assessed using the Mini-Mental State Examination (MMSE; Derouesne et al., 1999; Folstein et al., 1975). This 30-item scale assesses the severity of cognitive decline through items such as orientation, learning, attention, arithmetic, memory, language, and constructive praxis. The scale is rated from 0 to 30, reflecting the degree of cognitive impairment.

Physical function was assessed using the Short Physical Performance Battery (Guralnik et al., 1994, 2000). Three subtests measure lower-extremity functions: keeping balance (feet side-by-side, semi-tandem, tandem balance position, to be kept for 10 s each); walking 4 m at usual pace, yielding a gait speed ratio (meter per second); standing up and sitting down 5 times as quickly as possible with the arms folded. A cutoff of ≤8 points indicates poor physical performance (Cruz-Jentoft et al., 2019). Moreover, we used the item “activities” of the Braden scale (Bergstrom et al., 1987) to determine the percentage of residents confined to bed or in wheelchairs.

The validated French-language version of the Mini Nutritional Assessment—Short Form (MNA-SF) was used to assess the nutritional status of all patients (Lilamand et al., 2015). Each participant was evaluated based on the following: food intake, weight loss, mobility, psychological stress or acute disease, neuropsychological problems (only dementia), and body mass index. The final score was used to classify individuals as undernourished (0–7 points), at risk of undernutrition (8–11 points), or as having a normal nutritional status (13–14 points).

At last, the quality of life of the participant was assessed with the Quality of Life—Alzheimer’s Disease (QoL-AD) questionnaire (Logsdon et al., 2002; Wolak et al., 2009). This 13-item questionnaire assesses the participant’s physical condition, mood, relationships with friends and family, financial difficulties, and overall quality of life.

### KASEHPAD Study

KArukera Study of Aging in EHPAD (KASEHPAD) study is a twin study conducted in six French West Indies nursing homes (Guadeloupe and Martinique, French West Indies). This study has the same design (longitudinal cohort), the same follow-up, and the same objectives as the KASAF study. The trial was registered on ClinicalTrials.gov on October 13, 2020 (NCT04587466). In the six nursing homes, 332 residents aged 60 or older were included between September 2020 and November 2022. For the purpose of this study, we extracted the baseline sociodemographic characteristics (age, gender), ADL, and MMSE scores of the KASEHPAD cohort.

### Statistical Analysis

Quantitative variables were expressed as mean ± standard deviation (*SD*), median, and interquartile range (IQR). Qualitative variables were expressed as percentages. Missing values were not imputed. Fisher test and Wilcoxon’s tests were used to compare age, gender, ADL, and MMSE scores between residents in foster families and nursing homes. All analyses were performed with R v.4.2.1 software.

## Results

### Characteristics of Older Adults in Foster Families

**Figure 1.**
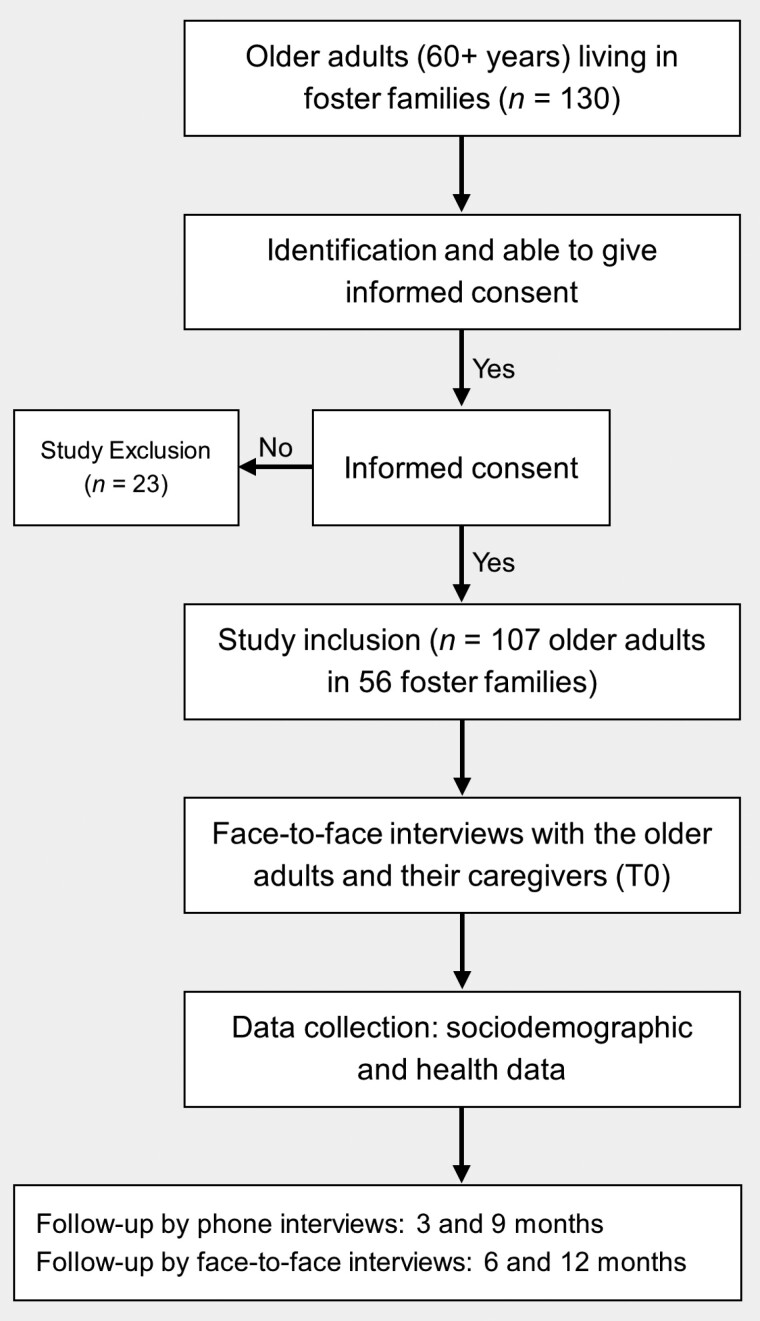
Flowchart of the KASAF study. KASAF = KArukera Study of Aging in Foster Families.

A total of 130 residents were identified in foster families and 107 residents (from 56 foster families) agreed to be included in the study (participation rate: 82.3%; Figure 1). The mean age was 81.8 ± 11.1 years (median 85 years). The majority were women (61.7%). They had lived in these families for a median of 3.2 years. Before entering foster families, half were living at home and 29.5% had been transferred from a hospital or a clinic. On entering in foster family, 17.9% of older adults were severely dependent, 70.6% were moderately dependent, and 11.3% were weakly dependent. Two thirds of the residents were single (Table 1) and 40 (40.0%) had no children. Within the foster family, almost all residents received daily nursing care, with one or two visits a day and 60% had a daily physiotherapist. Only two older adults smoked and two drank alcohol.

**Table 1.** Characteristics of Residents in Foster Families

Characteristics	Total (*n* = 107)
Age (years)	
Mean ± *SD*	81.8 ± 11.1
Median (min, max)	85 (60, 107)
% 75 years and older	74 (69.2%)
Gender (% men)	41 (38.3%)
Length of stay in foster families (years)	
Mean ± *SD*	4.8 ± 4.7
Median (min, max)	3.2 (0.1, 23.2)
Referral modality, *n* (%)	
Own home	52 (49.5%)
Hospital or clinic	31 (29.5%)
Nursing homes	8 (7.6%)
Other	14 (13.3%)
Functional level on entering foster family, *n* (%)	
GIR 1–-2 (severe dependency)	19 (17.9%)
GIR 3–4 (moderate dependency)	75 (70.6%)
GIR 1–2 (low dependency)	12 (11.3%)
Marital status, *n* (%)	
Widowed	22 (20.8%)
In a relationship	12 (11.3%)
Single or divorced	72 (67.9%)
Number of older adults per family, *n* (%)	
1 older adult	16 (28.5%)
2 older adults	28 (50.0%)
3 older adults	12 (21.5%)
Daily nursing care (= yes), *n* (%)	103 (96.3%)
Frequency (number per week), mean ± *SD*	12.5 ± 2.9
Physical therapy session (= yes), *n* (%)	65 (60.7%)
Frequency (number per week), mean ± *SD*	8.0 ± 2.5

*Note*: *SD* = standard deviation.

In terms of comorbidities, more than a quarter of the residents suffered from diabetes and almost half (45.8%) had hypertension, 14% had heart failure, 19.6% had a history of stroke, and 13.1% were hemiplegic. Half of the residents were considered to have a dementia, and 12% had Parkinson’s disease. Dementia was more common in women (60.0%) than in men (33.3%). There was only one case of cancer (prostate cancer in 2008; Table 2).

**Table 2.** Comorbidities of Older Adults Living in Foster Families

Comorbidities	*n* (%)
Diabetes	26 (25.5%)
Hypertension	49 (45.8%)
Considered to have a dementia	53 (49.5%)
Parkinson disease	13 (12.1%)
Heart diseases (myocardial infarction, congestive heart failure, angina)	16 (15.0%)
Stroke	21 (19.6%)
Hemiplegia	14 (13.1%)
Chronic obstructive pulmonary disease	2 (1.9%)
Arthritis	5 (4.7%)
Cancer history	1 (0.9%)
Depressive symptomatology	31 (29.0%)
Vision difficulties	30 (28.0%)
Hearing difficulties	11 (10.3%)

Among the 96 participants who completed the MMSE test, the mean score was 8.5 ± 10.2. Of these participants, 76% had a MMSE score <18. The mean (1.5 ± 1.79) and median (= 1) scores on the Katz ADL were low, and 33 residents had an ADL score of 0, corresponding to severe dependency. The most common dependency patterns were full assistance need with dressing (82.2%), bathing (79.4%), inability to use the toilet (69.1%), and incontinence (66.3%). The MNA-SF score was 9.3 ± 2.9, with 30 (28.0%) residents classified as malnourished (MNA score ≤7). Over 40% of participants were confined to a bed or a wheelchair. The prevalence of physical impairment (SPPB < 8) was 97.2% (Table 3).

**Table 3.** Scores at the Geriatric Scales of Older Adults Living in Foster Families

Scale	Mean ± *SD*	Median; IQR	*n* (%)
MMSE score (*n* = 96)	9.3 ± 10.1	0.0; 18	
Intellectually incompetent to pass the test or MMSE score = 0			53 (55.2%)
MMSE score 1–9			3 (3.1%)
MMSE score 10–18			17 (17.7%)
MMSE score >18			23 (23.9%)
ADL score (*n* = 107)	1.51 ± 1.79	1; 2.5	
ADL score 5–6			10 (9.3%)
ADL score 3–4			15 (14.0%)
ADL score 0.5–2			47 (43.9%)
ADL score = 0			33 (30.8%)
Needing full assistance for bathing			85 (79.4%)
Needing full assistance for dressing			88 (82.2%)
Needing full assistance for toileting			74 (69.1%)
Needing full assistance for transferring			42 (39.2%)
Incontinence			71 (66.3%)
Total feeding dependence			56 (52.3%)
IADL score (*n* = 106)	0.46 ± 0.87	0; 1	
IADL score = 0			72 (67.9%)
IADL score = 1			26 (24.3%)
IADL score 2–3			5 (4.7%)
IADL score 4–5			3 (2.8%)
MNA-SF score (*n* = 107)	9.3 ± 2.9	9; 4	
QoL-AD score (*n* = 47)	27.2 ± 8.8	26; 14.5	
SPPB score (*n* = 107)	1.0 ± 2.0	0; 1	
SPPB score <8			104 (97.2%)
Confined to bed or a wheelchair (Mobility Braden scale; *n* = 107)			45 (42.0%)

*Notes*: ADL = activities of daily living; IADL = instrumental activities of daily living; MMSE = Mini-Mental State Exam; MNA = Mini Nutritional Assessment—Short Form; QoL-AD = Quality of Life—Alzheimer’s Disease; *SD* = standard deviation; SPPB = Short Physical Performance Battery.

### Characteristics of Foster Caregivers

The mean age of the foster caregivers was 60.3 ± 5.5 years. The youngest was 46 and the oldest was 75. More than half (53.6%) were over 60 years old. Only one foster caregiver was male, all the others were female (98.2%). They had 2.5 ± 1.4 children. Almost 30% of the foster caregivers lived alone. The caregivers had been in the profession for 11.7 ± 7.3 years. More than half of the foster caregivers (54.7%) had already worked in the care sector before. The mean number of older adults cared for by each caregiver was 2.2 ± 0.6.

### Comparison of Characteristics of Older Adults Living in Foster Families or Nursing Homes

In French Caribbean nursing homes, the mean age of KASEHPAD participants was 81.3 ± 10.1 years and a half were men (50.5%). The mean ADL score was 2.40 ± 2.11 and the mean MMSE score was 11.27 ± 9.40. There was no difference in terms of age (*p* = .581) between residents in foster families and nursing homes. Older adults in foster families were less frequently men (38.3% vs 50.6%, *p* = .034) and presented a lower ADL score (1.5 ± 1.8 vs 2.4 ± 2.1, *p* < .001) and a lower MMSE score (9.3 ± 10.1 vs 11.3 ± 9.4; *p* = .028; Table 4).

**Table 4.** Comparison of Age, Gender, ADL, and MMSE Score Between Residents in Foster Families and Nursing Homes

Characteristics	Older adults in foster families (KASAF)			Older adults in nursing homes (KASEHPAD)			*p*
	Total	Mean ± *SD*	*n* (%)	Total	Mean ± *SD*	*n* (%)	
Age	107	81.8 ± 11.1		332	81.3 ± 10.1		0.581
Gender (men)	107		41 (38.3%)	332		168 (50.6%)	0.034
ADL score	107	1.5 ± 1.8		326	2.4 ± 2.1		<0.001
MMSE score	94	9.3 ± 10.1		295	11.3 ± 9.4		0.028

*Notes*: ADL = activities of daily living; KASAF = KArukera Study of Aging in Foster Families; KASEHPAD = KArukera Study of Aging in EHPAD; MMSE = Mini-Mental State Exam; *SD* = standard deviation.

## Discussion and Implications

In French West Indies, the results of the KASAF study on inclusion showed that foster families take in older adults with high levels of dependency. Indeed, the residents had comorbidities similar to those usually observed in nursing homes, especially in terms of dementia and autonomy. In our study, almost half of the residents were considered to have dementia. This rate of dementia is similar to that observed in nursing homes in the United States (Joyce et al., 2018). Nevertheless, based on the MMSE score, we observed that 76% of the participants had moderate to severe cognitive impairment (MMSE <18). Dementia is therefore likely to be underdiagnosed and undertreated in foster families. In addition, 40% of residents were confined to bed or a wheelchair. Autonomy scores were particularly low, with a median ADL score of 1, and two thirds of patients had an IADL score of 0. Numerous comorbidities were reported. Compared with a recent study in French nursing homes (Couderc et al., 2021), we found a higher rate of diabetes (17.9% vs 25.5% in our study), a lower rate of hypertension (53.5% vs 45.8%), and a lower rate of heart disease. Only one case of cancer history was observed in our study, whereas the overall prevalence of cancer in nursing homes is estimated at around 8% (Liuu et al., 2019). It is likely that older adults with a history of cancer or active cancer are therefore not referred to foster families, either by health professionals or their families. The clinical profile of foster families’ residents also presented several particularities. We observed a high proportion of men (39%), whereas the proportion of men in nursing homes is generally around 30% in France. We also found a high percentage of diabetes and Parkinson’s disease in this area, which have already been described in the literature (Carrère et al., 2018, Lannuzel et al., 2018) in these territories. Finally, we found that 40% of the older participants did not have children. Social isolation may partly explain their desire to move to a foster family rather than a nursing home.

In the French West Indies, foster families are considered as an alternative to nursing homes. Indeed, we observed that the clinical profile of foster family residents was quite similar to that observed of French Caribbean nursing home residents in terms of age, gender, dependency, and cognition. In fact, foster family residents appear to be more dependent and more cognitively impaired than nursing home residents. We noted that in the two accommodations, there was a high proportion of male and that residents were younger than those observed in mainland nursing homes (i.e., 86.1 years old; Balavoine, 2022). These particularities need to be investigated.

In the life course of older adults, the foster family is perceived as care for dependency in Guadeloupe, rather than care for frailty. There is a strong need for affordable alternatives to nursing homes and, therefore, for policies to provide such alternatives (Achou et al., 2022). The coronavirus disease (COVID) crisis highlighted the vulnerability of nursing home residents in the context of an epidemic. Strict containment and isolation measures to limit the spread of the virus and the overloading of emergency and resuscitation services have severely affected residents and caregivers in residential settings. In Canada, for example, 72% of people are less likely to enter a nursing home because of the pandemic (Achou et al., 2022). Foster families could be an interesting alternative for dependent older adults. As the clinical profiles of nursing home and foster family residents are similar in terms of dependency, the decision to move into a foster family could be a response to a social need. In a qualitative study (Chammem et al., 2021), “foster families were considered as more spontaneous and family-oriented structures with negotiable rules, offering personalized and close support in the face of loss of autonomy.” Indeed, the links between the foster careers and the care recipient are strong; the host family takes care of only one to three older adults. Residents’ participation in the family life and close contact with a single reference person could have a strong impact on their well-being and feelings of loneliness. In French nursing homes, the staffing ratio would be 70 per 100 beds, including 30 nursing staff (Boucaud-Maitre, Meillon et al., 2023). However, staff are responsible for separate tasks, such as cooking or administering medication, and are not always aware of a person’s needs and wishes. In foster families, residents are seen first as people, not patients, with medical care provided by professionals who come to monitor and treat residents on a daily basis. The main limitation of this Caribbean model is the strong commitment of the caregiver, for whom there are currently no support solutions or alternatives in case of holidays or illness. Extrapolation of this model to developed countries would probably require some adjustments, such as a time relay in nursing homes when necessary.

This study therefore provides interesting and new data on the profile of foster families’ residents in Guadeloupe and on the prevalence of geriatric syndromes. To our knowledge, this model of foster family for dependent older adults, with external medical supervision, has never been studied. The 1-year data from the KASAF study will help us to assess the foster family model, and to adapt the training of local stakeholders, as well as the provision of care and its organization at the local level.

This study presents limitations. We had initially planned to recruit 250 foster family residents, based on the number of foster families in Guadeloupe identified by the authorities and the possibility of each family to take in three older adults. In the end, we were only able to include 107 of the 130 older people we identified. Many of the registered foster families were no longer in activity, while others have turned to caring for people with disabilities. Finally, the inclusion and follow-up of participants took place during the COVID period. It cannot be excluded that some of the variables studied were affected by the COVID crisis, in particular deaths, hospitalizations, and psychological outcomes.

In conclusion, long-term care systems vary between countries, reflecting available resources and existing infrastructures, including health services and cultural considerations. Foster families for older adults might represent an interesting strategy to address the unmet clinical and social needs. The inclusion data from our study suggest that foster families in Guadeloupe care for older adults who match the clinical profile of patients in nursing homes, except for older adults with cancer. Assessing this model is therefore important, as it could meet a key need for many countries that have not developed, or do not wish to develop, nursing home models.

## Data Availability

The study was preregistered on the Clinical Trials Registry (NCT04545775). The data sets generated and/or analyzed during the current study are not publicly available. Authors have not completed their original work with the data set.
